# Engaging community pharmacists in tuberculosis-directly observed treatment: a mixed-methods study

**DOI:** 10.1017/S1463423623000105

**Published:** 2023-03-22

**Authors:** Yen Jun Wong, Chern Choong Thum, Khuen Yen Ng, Shaun Wen Huey Lee

**Affiliations:** 1 School of Pharmacy, Monash University Malaysia, Jalan Lagoon Selatan, 47500 Subang Jaya, Selangor, Malaysia; 2 Department of Psychiatry, Hospital Sultan Abdul Aziz Shah Universiti Putra Malaysia, Persiaran Mardi – UPM, 43400 Serdang, Selangor, Malaysia; 3 School of Pharmacy, Taylor’s University Lakeside Campus, Jalan Taylors, 47500 Subang Jaya, Selangor, Malaysia; 4 Center of Global Health, Perelman School of Medicine, University of Pennsylvania, Philadelphia, PA, USA

**Keywords:** community pharmacist, directly observed treatment, mixed-methods study, public-private mix, tuberculosis

## Abstract

**Aim:**

This study aimed to evaluate the feasibility of implementing community pharmacy-based tuberculosis-directly observed treatment (TB-DOT) in Malaysia.

**Background:**

Tuberculosis (TB) eradication is one of the top priorities in the public health agenda in Malaysia. While public-private mix (PPM) initiatives have been launched, community pharmacists remain undervalued assets in TB management.

**Methods:**

A two-phase mixed-methods study targeting community pharmacists was conducted in Malaysia between March and October 2021. The first phase was an online self-administered survey developed according to the Consolidated Framework for Implementation Research (CFIR). The second phase was a semi-structured interview to allow deeper understanding on the quantitative results. Quantitative data were analysed using descriptive analysis while qualitative data were analysed using thematic analysis with a semi-inductive approach. The data were triangulated to enhance comprehensiveness and credibility of the findings.

**Findings:**

The survey was completed by 388 community pharmacists, and 23 pharmacists participated in the interview. Most community pharmacists indicated their willingness to serve as TB-DOT supervisors (70.1%). Qualitative results supported the findings. Community pharmacy-based TB-DOT service was perceived as an avenue to improve TB management and outcomes and to enhance the professional role of pharmacists in TB service at primary care settings. This was also perceived as a feasible intervention with the potential to strengthen the National TB Control programme. This initiative needs be reinforced with adequate support from the public healthcare sector for a strong partnership in ensuring success.

## Introduction

In the late 1800s, tuberculosis (TB) was one of the leading causes of mortality, accounting for one in every seven deaths in Europe and the United States (Centers for Disease Control and Prevention, 2016). With social and economic development alongside the discovery of effective drug treatment, the number of TB cases has declined significantly. While TB diagnosis and treatment had successfully prevented 66 million deaths in the last two decades, TB still affects 10.6 million people worldwide, resulting in 1.6 million deaths in 2021 (World Health Organization, 2022).

In addressing the global TB epidemic, the directly observed treatment-short course (DOTS) programme has been used as a TB control strategy (World Health Organization, 1999). The DOTS programme combines five components: government commitment, case detection by sputum smear microscopy, regular drug supply, a standardized recording and reporting system, and the TB-directly observed treatment (TB-DOT). TB-DOT is a process where TB patients consume their daily medications under the supervision of a qualified healthcare worker for the first two months of treatment (World Health Organization, 1999). While DOTS programme is a cost-effective approach to manage TB patients, treatment failure due to non-adherence is still an issue (Tola *et al*., 2015). This can be attributed to socioeconomic and behavioural factors, including the lack of transportation, cost, social support, and poor communication between patients and health care workers (Tola *et al*., 2015; Cai *et al*., 2015). In response to these barriers, multi-sectorial partnerships for the Public-Private Mix (PPM)-DOTS have been suggested (Malmborg *et al*., 2011; Uplekar, 2003). The PPM-DOTS is also known as the ‘PPM for TB Care and Control’ (Malmborg *et al*., 2011).

Studies have shown that TB management through PPM is a substantially accessible and supportive platform for patients to receive TB service especially in resource limited settings (Lei *et al*., 2015; Malmborg *et al*., 2011). With improved patient accessibility, this led to increased case detection and better treatment outcomes. For example, the private practitioners in India, Indonesia, Bangladesh, and the Philippines had contributed to TB management in the primary care settings (Lei *et al*., 2015; Malmborg *et al*., 2011). In addition, evidence has also shown the implementation of TB-PPM in several low-middle income countries (LMICs), namely Cambodia, Myanmar, and Vietnam had strengthened the collaboration between the public and private sectors in facilitating the referral, TB screening, and diagnosis (Bell *et al*., 2015; Lonnroth *et al*., 2003; Mihalea and Richardson, 2011; Thu, 2015; Wong *et al*., 2022; Zawahir *et al*., 2021). However, community pharmacists, as healthcare providers from the private sector, are underutilized in the delivery of TB service, in particular TB-DOT (Konduri *et al*., 2017; Lei *et al*., 2015).

Malaysia currently has an estimated TB incidence rate of 92 per 100, 000 population, with around 80% of treatment success rate. The TB burden and treatment success rate had not improved over the past five years (World Health Organization, 2021). To meet the ambitious milestone of ending TB epidemics by 2035, imperative efforts are required to provide an equitable access of high-quality TB diagnosis, treatment, care, and prevention to all levels of communities (Stop TB Partnership, 2015; World Health Organization, 2015). The participation of community pharmacists in TB management could be an untapped opportunity for Malaysia.

Studies to date have reported that community pharmacists-led interventions have successfully improved treatment adherence and disease outcomes. Some of these services include the medication review (Al-babtain *et al*., 2022; Jokanovic *et al*., 2017) and smoking cessation services (Carson-Chahhoud *et al*., 2019). In these pharmaceutical care services, community pharmacists focus on person-centred care and patient engagement. This enables drug and ailment-related concerns, and patients’ unmet needs to be addressed promptly, leading to positive outcomes in their health management.

Incorporating community pharmacists in the PPM remains a novel concept in Malaysia. However, this could potentially be an avenue to enhance TB management and health outcomes of the TB-affected communities. Therefore, it is important that we explore the barriers and facilitators at the pre-implementation stage, in order to better facilitate the integration of TB-DOT service as part of community pharmacists’ professional practice. This study aimed at evaluating the feasibility of implementing TB-DOT service at community pharmacies in Malaysia.

## Methods

### Study design

This mixed-methods study was conducted online between March and October 2021. The survey was conducted from March to August 2021, while the semi-structured interviews took place from September 2021 to October 2021 after the survey period ended to gather additional insights based on the results from the survey. The study consisted of two phases: Phase 1 where participants answered an online self-administered questionnaire and Phase 2 which involved an online one-on-one semi-structured interview. The study was approved by the Monash Health Human Research Ethics Committee (Project ID: 27294).

### Phase 1 self-administered survey

#### Questionnaire

A questionnaire was developed based on TB guidelines and published literature using the Consolidated Framework for Implementation Research (CFIR) (Supplementary material S1 and S2) (Damschroder *et al*., 2009; Ministry of Health Malaysia, 2012). This implementation science framework was selected as it could guide continuous evaluation of an evidence-based intervention including both the pre-implementation and post-implementation stages of a pharmaceutical service. It consists of a comprehensive listing of constructs that aims at identifying potential factors affecting the intervention. This allows researchers to improve constructs that affect the process of implementation.

Nine constructs from four CFIR domains were selected to evaluate the feasibility of implementing community pharmacy-based TB-DOT service in Malaysia. The definitions and reasons of selecting the CFIR domains and constructs are described in Table 1. The survey questions were then developed according to the selected constructs in English.

**Table 1.** The Consolidated Framework for Implementation Research (CFIR) domains and constructs selected in developing the study

CFIR domains	CFIR construct	Short description of CFIR construct (16)	Reasons of selecting the CFIR construct
A. Inner setting			
	A1. Pharmacy characteristics	The social architecture, age, maturity, and size of an organization.	To understand the structure and the availability of manpower for the facility.
	A2. Implementation climate	The absorptive capacity for change, shared receptivity of involved individuals to an intervention, and the extent to which use of that intervention will be rewarded, supported, and expected within their organization.	To identify the receptivity of the pharmacists in implementing the intervention.
B. Outer setting			
	B3. Patients’ needs and resources	The extent to which patient needs, as well as barriers and facilitators to meet those needs, are accurately known and prioritized by the organization.	To identify patients’ challenges, gaps, and need in TB care.
	B4. External policies and incentives	A broad construct that includes external strategies to spread interventions, including policy and regulations (governmental or other central entity), external mandates, recommendations and guidelines, pay-for-performance, collaboratives, and public or benchmark reporting.	To understand the need of guidelines and incentives for pharmacists in implementing the intervention.
C. Intervention characteristics			
	C5. Relative advantage	Stakeholders’ perception of the advantage of implementing the intervention versus an alternative solution.	To identify pharmacists’ perception on the advantage of implementing the intervention.
	C6. Complexity	Perceived difficulty of the intervention, reflected by duration, scope, radicalness, disruptiveness, centrality, and intricacy and number of steps required to implement.	To identify perceived concerns to be addressed before the implementation of the intervention.
	C7. Adaptability	The degree to which an intervention can be adapted, tailored, refined, or reinvented to meet local needs.	To identify if pharmacists have relevant experience to adopt and adapt the intervention.
D. Characteristics of Individuals			
	D8. Knowledge and beliefs about the medical condition and interventions	Individuals’ attitudes towards and value placed on the intervention as well as familiarity with facts, truths, and principles related to the intervention.	To understand pharmacists’ knowledge and understanding of TB and its management.
	D9. Self-efficacy	Individual belief in their own capabilities to execute courses of action to achieve implementation goals.	To understand pharmacists’ confidence in implementing the intervention.

CFIR = Consolidated Framework for Implementation Research; TB = tuberculosis.

Content validity of the questionnaire was evaluated by an expert panel comprising 12 international and local experts with more than 10 years of experience from community pharmacies, pharmacy research, and TB research. Following comments from the experts, amendments were made on the structures and flow of the questions. The questionnaire was then pilot-tested in 10 community pharmacists for face validity before the study recruitment. Five additional questions were posted at the end of the survey, to receive feedback from the participants on the suitability, appropriateness, relevance, clarity, and usefulness of the questionnaire. All agreed that the questionnaire fulfilled the abovementioned criteria without further comment.

The final questionnaire comprised 42 questions, including demographic information. The questions were a mixture of multiple-choice structures, with a binary response (‘Yes’, ‘No’), multiple-choice list, Likert-type scale, order ranking, checkboxes (where participants could select multiple answers from the available choices), and open-ended questions to collect the demographic data (Supplementary material S2). For questions with binary response and factual questions, responses of ‘Yes’ or correct answers were coded with 1, whereas ‘No’ or wrong answers were coded with 0. For questions with multiple-choice list, checkboxes, order ranking and Likert scale, each response option was pre-coded with a numerical value, for example ‘Extremely willing = 5; Very willing = 4; Moderately willing = 3; Slightly willing = 2; Not willing at all = 1’.

#### Data collection

In the year of 2021 when the study was conducted, there were approximately 6,000 pharmacists practising in the private sector for the entire country of Malaysia (Pharmacy Board Malaysia, 2022; Pharmacy Services Programme, 2022; Pharmacy Services Programme, 2021). Assuming a margin of error of 5%, confidence level of 95%, and 5% drop-out (Dong and Peng, 2013; Schafer, 1999), a minimum sample size of 380 participants was required (Supplementary material S3). Participants were recruited through convenience sampling. Invitations were sent through emails, the Malaysian Pharmacists Society electronic newsletter, and social media (Facebook, Twitter, and LinkedIn). Fifteen fortnightly reminder emails and social media posts were sent out to increase the response rate. A total of 6330 study invitations, including reminders, were sent via emails. To ensure the sample could better represent the population of practising community pharmacists in Malaysia, 500 paper copies of the study invitation were also sent to community pharmacists from the states with higher TB incidence rates and rural areas.

### Phase 2 semi-structured interview

The qualitative study was explanatory in nature and built upon the Phase 1 quantitative study. The semi-structured interview was conducted after the quantitative data collection. The topic guide was developed based on a priori on the CFIR domains and preliminary survey results that enabled the researchers to have a deeper understanding of the quantitative data during the interview (Supplementary material S4).

Convenience sampling was applied to recruit participants. The inclusion criteria for participant recruitment include practising community pharmacists in Malaysia who agreed to participate in the interview. Moreover, the interviewer should have no acquaintance with the interviewee. The interview invites and the link directing participants to a separate sheet of sign-up form were posted at the end of the online survey. Participants who signed-up were contacted by the researcher (Author 1) via email to schedule the interview. The interview was conducted by Author 1 and supervised by Author 4, through phone call or video call using Zoom. The interviews averaged 44 min (range: 26 to 63 min). All interview sessions were audio-recorded and transcribed verbatim. The recordings and transcripts were de-identified. Data collection and analysis were carried out concurrently. The first five interviews were used as the base case with two interview sessions for each subsequent run. Recruitment continued until data saturation where there was no new information emerged. Data saturation was achieved in the twenty-third interview with 23 participants in total.

### Data analysis

Quantitative data were analysed using IBM SPSS Statistics version 28.0 (IBM Corporation, Armonk, NY, USA). Data were presented as mean with standard deviation (SD), median with interquartile range (IQR), or frequency with percentages (%). In the event of incomplete demographic data, these were excluded from the analysis.

Qualitative data were analysed using QSR Nvivo version 20.3 (QSR International Pty Ltd, Burlington, MA, USA). The data were analysed using thematic analysis with a hybrid approach of deductive and inductive methods (Fereday and Muir-Cochrane, 2006). This hybrid method was applied as the deductive approach allowed codes to be mapped into core themes derived from the CFIR domains. Where appropriate, the inductive approach could also allow the enrichment of findings if there were new themes and subthemes to be derived from the codes. The transcripts were coded after each interview. Each transcript was cross-checked and read repeatedly by the researchers (Author 1 and Author 2) to identify the code pattern. A coding framework was developed by Author 1 and then reviewed by Author 2 through pre-testing for validation. Subsequent coding process was conducted by Author 1 and guided by the coding framework developed. A total of 132 codes were generated from the transcripts. Both authors discussed on the codes generated, and 22 (16.7%) of the codes were re-coded or merged, resulting in 110 codes in total. The codes were then organized into themes aligned with a priori CFIR domains by Author 1 and cross-checked by the team members for consensus.

Information trustworthiness was established through methodological triangulation of both results from the quantitative survey which was validated in our semi-structured interview. Through this method, we could conduct a comparison of results between the quantitative and qualitative findings to enhance data credibility. Constant discussions among the team members also facilitated the process of refining the organization of the codes.

## Results

### Participants’ characteristics

A total of 624 participants responded to the survey, and 388 of them completed the survey. Among the incomplete survey, 111 had only responded to the electronic consent form, and the remaining 125 had fully or partially completed the demographic details, but they did not progress further to the next session of the questionnaire. The participants had a mean age of 31.2 ± 6.8 years old with 5.0 ± 6.2 years of working experience as a community pharmacist (Table 2). Another 23 participants completed the interview. The interviewees had a mean age of 29.9 ± 3.9 years old with 5.2 ± 4.1 years of experience practising as a community pharmacist.

**Table 2.** Demographics of participants

Participants’ characteristics	Survey, n (%) (N = 388)	Interview, n (%) (N = 23)
Age (years old)		
Mean ± SD	31.2 ± 6.8	29.9 ± 3.9
Median (IQR)	29.0 (27.0 – 33.0)	29.0 (28.0 – 31.5)
Range	23.0 – 69.0	25.0 – 39.0
Gender		
Female	267 (68.8)	16 (69.6)
Years of practice (years)		
Mean ± SD	5.0 ± 6.2	5.2 ± 4.1
Median (IQR)	3.0 (1.0 – 6.25)	4.0 (3.0 – 6.5)
Range	0.08 – 38.0	1.0 – 16
≤5 years	282 (72.7)	16 (69.6)
>5 years	105 (27.1)	7 (30.4)
Missing data	1 (0.2)	–
Previous working experience in the Government sector		
Yes	161 (41.5)	12 (52.2)
No	227 (58.5)	11 (47.8)
Experience in specialized pharmacy service*		
Yes	122 (31.4)	14 (60.9)
Smoking cessation	65 (53.3)	10 (43.5)
Diabetes management	35 (28.7)	3 (13.0)
Others^#^	24 (19.7)	7 (30.4)
Location of pharmacy		
Urban	254 (65.5)	15 (65.2)
Suburban	113 (29.1)	4 (17.4)
Rural	21 (5.4)	4 (17.4)
Pharmacy setting		
Shop lot	309 (79.6)	18 (78.3)
Others (in shopping mall, health facilities, or office)	79 (20.4)	5 (21.7)
Ownership		
Chain	193 (49.7)	14 (60.9)
Franchise, independent or others	195 (50.3)	9 (39.1)
Average number of customers per day		
≤100	235 (60.6)	14 (60.9)
>100	150 (38.7)	9 (39.1)
Missing data	3 (0.7)	–
Number of fully registered pharmacists per shift		
≤2	308 (79.4)	18 (78.3)
>2	79 (20.4)	5 (21.7)
Missing data	1 (0.2)	–
Number of pharmacy assistants or staff per shift		
≤5	288 (74.2)	19 (82.6)
>5	96 (24.7)	4 (17.4)
Missing data	4 (1.1)	–
Availability of private consultation room		
Yes	181 (46.6)	9 (39.1)
No	207 (53.4)	14 (60.9)

SD = standard deviation; IQR = interquartile range; * Respondents could have experience in more than one specialized pharmacy service; ^#^Other specialized pharmacy services include retroviral disease management, lungs disease management, methadone replacement therapy, skin/wound care, pain management, warfarin adherence monitoring and management, compounding.

### Phase 1 quantitative findings

The perceived facilitators and barriers in adopting community pharmacy-based TB-DOT service based on the CFIR domains and constructs were evaluated and described as follows (Table 3).

**Table 3.** Findings from the phase 1 survey

CFIR domain	Question number	Questions	Responses, n (%)
A. Inner setting			
Pharmacy characteristics	11	How many full-time fully registered pharmacists work at this community pharmacy per shift?	
		• 1 to 2	308 (79.4)
		• More than 2	80 (20.6)
	12	How many pharmacy assistants and staff work at this community pharmacy per shift?	
		• None	6 (1.5)
		• 1 to 4	216 (55.7)
		• More than 4	166 (42.8)
	13	Does this community pharmacy have a private consultation room?	
		• Yes	181 (46.6)
		• No	207 (53.4)
Implementation climate			
	21	Community pharmacists in Malaysia may have limited role in TB management. However, in your opinion, how could a community pharmacist contribute to TB management? (Select all that apply)	
		• Refer customers with suggestive symptoms of active pulmonary TB to doctors or specialists for thorough examination	346 (89.2)
		• Educate public and patients on tuberculosis prevention practice	338 (87.1)
		• Monitor side effects of the TB medications and refer patients to doctors or specialists when necessary	304 (78.4)
		• TB medication counselling	292 (75.3)
		• Review medication list of patients to identify any possible drug interactions	289 (74.5)
		• Observe and witness patients or clients taking TB medications (TB-directly observed treatment, TB-DOT)	211 (54.4)
		• Send reminder to TB patients to ensure their adherence in taking TB medications	197 (50.8)
		• Maintain records of visits by checking TB patients who are lost to follow-up	174 (44.8)
	27	Are you willing to introduce and provide TB-DOT to TB patients or customers who may need it?	
		• Yes	272 (70.1)
		• No	116 (29.9)
	28	If yes, you would be happy to provide TB-DOT for TB patients in which phase of TB treatment? Note: Drug-sensitive TB disease treatment regimen consists of an initial 2-month intensive phase followed by a maintenance phase of at least 4 months.	
		• Intensive phase only (at least 2 weeks after the initiation of TB treatment, when it is not contagious)	25 (9.2)
		• Maintenance phase only	162 (59.6)
		• Both intensive and maintenance phases	85 (31.3)
	36	It is acknowledged that only a limited number of community pharmacies in Malaysia provide drive-through service, due to substantial restrictions. Nevertheless, in your opinion, do you think that providing TB-DOT by drive-through could be a practical alternative?	
		• Yes	210 (54.1)
		• No	178 (45.9)
	37	In your opinion, do you think that monitoring TB-DOT through video call (video DOT) by community pharmacists could be a practical alternative?	
		• Yes	190 (49.0)
		• No	198 (51.0)
	38	If you are an employee to your community pharmacy, do you think your employer would be supportive to implement DOTS (including TB-DOT) as part of community pharmacists’ daily practice? OR If you are an employer, would you be supportive to implement DOTS (including TB-DOT) as part of community pharmacists’ daily practice?	
		• Yes	111 (28.6)
		• No	78 (20.1)
		• Unsure	199 (51.3)
B. Outer setting			
Patients’ needs and resources	22	Have you ever referred patients or customers with suggestive symptoms of active pulmonary TB for medical screening and examination from your community pharmacy for the past one year?	
		• Yes	120 (30.9)
		• No	268 (69.1)
	23	Do you think there is a need for an official and structured referral system for referral?	
		• Yes	324 (83.5)
		• No	64 (16.5)
	24	How often does a patient or customer look for TB medications at your community pharmacy for the past one year?	160 (41.2)
		• More than once a month	96 (24.7)
		• Rarely (less than once per month)	64 (16.5)
		• Never	228 (58.8)
	25	Dispensing of TB medications at community pharmacies may not be a common practice in Malaysia. However, do you think there is a need of dispensing and re-filling TB medications at community pharmacies in Malaysia?	
		• Yes	288 (74.2)
		• No	100 (25.8)
	20	Based on your experience, what are the potential difficulties or challenges that TB patients face? (Please rank each of the following options in order of importance with #1 being the most important challenge to be addressed, to #10 being the least important challenge to be addressed). (Top 3 rankings were presented)	
		• Rank 1: Not adherent to medication consumption	154 (40.0)
		• Rank 2: Experiencing side effects to medications	91 (23.6)
		• Rank 3: Unaware of the severity of TB disease	87 (22.6)
	26	TB-DOT at community pharmacies has not been implemented at community pharmacies in Malaysia. However, in your opinion, do you think there is a need for TB-DOT at community pharmacies in Malaysia?	
		• Yes	285 (73.5)
		• No	103 (26.5)
External policies and incentives	39	Do you think community pharmacists should receive reimbursement, remuneration or incentive for providing DOTS?	
		• Yes	354 (91.2)
		• No	34 (8.8)
		•	
	41	How effective do you think audit for TB service at community pharmacies is able to improve the quality of care and treatment adherence of TB patients?	383 (98.7)
		• Extremely effective	43 (11.1)
		• Very effective	154 (39.7)
		• Moderately effective	166 (42.8)
		• Slightly effective	20 (5.2)
		• Not effective at all	5 (1.3)
	42	If DOTS is to be implemented at your community pharmacy, how willing are you to be committed to audit for the monitoring and regulations in providing TB service?	365 (94.1)
		• Extremely willing	25 (6.4)
		• Very willing	115 (29.6)
		• Moderately willing	185 (47.7)
		• Slightly willing	40 (10.3)
		• Not willing at all	23 (5.9)
	40	Based on your opinion, what aspects of the TB service at the community pharmacies are likely to be audited? (Select all that apply)	
		• Infection control	354 (91.2)
		• Training of the staff on infection control	340 (87.6)
		• Staff personal protective respiratory measures	311 (80.2)
		• Administrative measures	273 (70.4)
		• Environmental measures	271 (69.8)
		• Management of adverse events from TB treatment	245 (63.1)
		• Treatment outcomes	232 (59.8)
C. Intervention characteristics			
Relative advantage	30	In your opinion, how would TB-DOT at community pharmacies benefit the TB patients? (Select all that apply) (Top 3 rankings were presented)	
		• Time flexibility	325 (83.8)
		• Better accessibility	312 (80.4)
		• Lesser waiting time spent to receive TB-DOT	305 (78.6)
Complexity	35	Do you think TB-DOT at community pharmacies is too complicated to be implemented?	
		• Yes	283 (72.9)
		• No	105 (27.1)
	34	What are the potential concerns you have towards DOTS at community pharmacies? (Please rank each of the following options in order of the degree of concern, with #1 being the most concerned barrier, to #10 being the least concerned barrier. (Top 3 rankings were presented)	
		• Rank 1: Unsure about the regulation requirements	287 (74.0)
		• Rank 2: Community pharmacists would be overly burdened by the workload of delivering TB-DOT	217 (55.9)
		• Rank 3: Stigma and pharmacy sales affected	199 (51.3)
	31	In your opinions, do you think delivering TB-DOT at community pharmacies could put the health of community pharmacists, pharmacy staff and customers at risk, considering the risk of an airborne disease?	
		• Yes	306 (78.9)
		• No	82 (21.1)
	32	In your opinion, do you think your pharmacy needs to be upgraded to accommodate the delivery of TB-DOT, for example to establish a private consultation room, counter shield etc?	
		• Yes	273 (70.4)
		• No	115 (29.6)
	33	Are you willing to invest in additional special kits or personal protective equipment (PPE) such as the N95 masks to deliver TB-DOT?	
		• Yes	232 (59.8)
		• No	156 (40.2)
Adaptability	6	Did you have experience working in the Government hospital or health clinic?	
		• Yes	161 (41.5)
		• No	227 (58.5)
	5	Have you been certified to provide specialized pharmacotherapy services?	
		• Yes	122 (31.4)
		• No	266 (68.6)
D. Characteristics of individuals			
Knowledge and beliefs about the interventions	15	To your knowledge, is there a National Tuberculosis Control Programme (NTP) in Malaysia?	
		• Yes	274 (70.6)
		• No	114 (29.4)
	16	What are the symptoms of active pulmonary TB? (Select all that apply)* (Correct answers were in bold)	
		• **Prolonged cough (more than 2 weeks)**	369 (95.1)
		• **Haemoptysis**	296 (76.3)
		• Gastric pain	45 (11.6)
		• **Night sweat**	283 (72.9)
		• **Loss of weight**	319 (82.2)
		• **Fever**	263 (67.8)
		• **Correctly identified all symptoms**	**150 (38.7)**
	17	What are the regimens used in treating active pulmonary TB in Malaysia according to the Malaysian Clinical Practice Guidelines? (Select all that apply)* (Correct answers were in bold)	
		• **Rifampicin**	370 (95.4)
		• Daptomycin	13 (3.4)
		• **Streptomycin**	70 (18.0)
		• **Ethambutol**	337 (86.9)
		• **Pyrazinamide**	315 (81.2)
		• **Isoniazid**	364 (93.8)
		• **Correctly identified all medicines used to treat active pulmonary TB**	**70 (18.0)**
	18	What are the side effects related to TB medications? (Select all that apply)* (Correct answers were in bold)	
		• **Discoloration of urine**	323 (83.2)
		• **Blurred vision**	230 (59.3)
		• **Hepatotoxicity**	326 (84.0)
		• **Joint pain**	162 (41.8)
		• **Correctly identified all potential side effects**	**97 (25.0)**
	19	At this point of time, do you know what to do when a TB patient experiences side effects due to TB medications?	
		• Yes	216 (55.7)
		• No	172 (44.3)
Self-efficacy	29	At this point of time, are you confident to provide TB-DOT at community pharmacies?	
		• Yes	207 (53.4)
		• No	181 (46.6)

CFIR = Consolidated Framework for Implementation Research; TB = tuberculosis; DOTS = directly observed therapy-short course; TB-DOT = tuberculosis-directly observed treatment; vDOT: video directly observed treatment; * According to the Malaysian Clinical Practice Guidelines – Management of Tuberculosis, 3^rd^ edition.

#### Inner setting

Most community pharmacies were often managed by one or two pharmacists (79.4%) and assisted by one to four staffs (55.7%) per shift. Approximately half of the community pharmacies also had a private consultation room (46.6%) which could be used for TB-DOT. Most participants believed that community pharmacists could contribute to TB referral (89.2%), TB education (87.1%), TB treatment side effect monitoring (78.4%), and TB medication counselling (75.3%). While only 54.4% of the participants believed community pharmacists could contribute as a TB-DOT supervisor, more than two-thirds of them were willing to introduce and provide TB-DOT to TB patients (70.1%). There was a mixed reaction on their willingness to provide TB-DOT through a drive-through service (54.1%) or via video DOT (vDOT, 49.0%). Only 28.6% of the participants were confident that their employers would be supportive of integrating DOTS as part of the community pharmacy-based pharmaceutical services.

#### Outer setting

Nearly one in three participants had encounters with customers who experienced TB symptoms requiring further referrals for TB screening over the past one year (30.9%). Since there was an absence of a systematic referral pathway in Malaysia, approximately 83.5% of the participants believed it is important to have an official and structured referral system to ensure all individuals with presumptive TB complete the medical screening for diagnosis.

While less than half of the participants had requests from customers for TB medications over the past one year (more than one request per month, 24.7%; rarely, 16.5%), almost three quarters believed there was a need to dispense TB medications at community pharmacies. Most participants also believed there was a need to provide TB-DOT at community pharmacies (73.5%) to address the challenges faced by TB patients during their treatment. The challenges include non-adherence to TB medications (40.0%), side effects resulting from TB medications (23.6%), and TB patients lacking awareness of the disease severity (22.6%).

Most participants believed pharmacists should receive reimbursement, remuneration, or incentives for providing DOTS at community pharmacies (91.2%). They also believed that audits would be moderately effective in monitoring the quality of TB service at the pharmacy (42.8%), and most were moderately willing to be audited for the monitoring and regulations in providing TB service (47.7%). They believed the scopes of infection control (91.2%), training of the staff on infection control (87.6%), and staff personal protective respiratory measures (80.2%) would most likely be audited.

#### Intervention characteristics

Participants agreed that community pharmacy-based TB-DOT would be beneficial to TB patients as it could provide better time flexibility (83.8%), better accessibility (80.4%) with lesser waiting time spent at the community pharmacies to receive TB-DOT compared to hospitals or clinics (78.6%).

Almost three quarters of them thought TB-DOT was difficult to implement (72.9%). The top three concerns were the regulation requirements (74.0%); workload of the pharmacists in delivering TB-DOT (55.9%); and stigma against TB patients which might affect pharmacy business (51.3%). Considering TB is an airborne infectious disease, there were also concerns that TB-DOT at community pharmacies that could put the health of people at the pharmacies at risk (78.9%). In view of the concerns, most participants believed the pharmacies require facility upgrade (70.4%), with private consultation room or counter shield to accommodate the delivery of TB-DOT. Approximately 59.8% of them were willing to invest in the personal protective equipment.

In evaluating the adaptability of this intervention, less than half of the participants reported having previous experience working in the Government healthcare facilities (41.5%) and were certified to provide specialized pharmacotherapy services (31.4%).

#### Characteristics of individuals

Most participants were aware of the National TB Control Programme (70.6%). Nevertheless, less than half of them were aware of all the symptoms (38.7%), all the approved regimens (18.0%), and side effects of TB medications (25.0%). Slightly more than half of them were confident to provide TB-DOT service (53.4%).

### Phase 2 qualitative findings

Findings from the survey were further explored in the interview. Quotes from the interviews are tabulated in Table 4.

**Table 4.** Quotes from participants during the phase 2 semi-structured interviews

CFIR domain and constructs	Statement	Quotes from participants
Inner setting		
A1. Pharmacy characteristics A2. Implementation climate	Statement 1	Some community pharmacists have patients literally lining up to see them… to counter that maybe a scheduled time, where they will come in, or set a time where, patient A is coming in every day at 7pm; patient B is coming in every day at a certain time. At least the pharmacists can anticipate when they are coming, can get someone to take over for them at the medication counter. (Community Pharmacist 20)
	Statement 2	We should have a separate consultation room, which is not through the main access… I’m sure that the patient will not let others to know. So if someone sees them walk in and out the pharmacy daily. It looks fishy. They can build another kind of consultation room, or drive-through, bypass. (Community Pharmacist 19)
	Statement 3	But video call … some people don’t have internet, or maybe some people just don’t have a smartphone. (Community Pharmacist 12)
	Statement 4	We can do a TB awareness programme, maybe and have some brochure, share the symptoms of TB, what are the alarming symptoms that they really should go to see a doctor, before things get worse. (Community Pharmacist 12)
	Statement 5	My boss is very supportive to what we do. So if you suggest something, which benefits the patients in long run … like, helping the community, go ahead … Because business is not just about profit. So as long as you help the people, business will come afterwards. (Community Pharmacist 11)
Outer setting		
B3. Patients’ needs and resources	Statement 6	So much medication, [with] long-term side effects. So of course, when they feel better, they will just no need to continue [treatment]… So, let’s say if they can get a nearby community pharmacy, of course, we can give some advice to them. I do think it will contribute to the success of TB medication completion rates. (Community Pharmacist 12)
	Statement 7	You can help them to, you can monitor their treatment regimen, and make sure that there’s nothing wrong, or there’s any concern that the patient has, you can answer them all, if not direct them, to refer them back to the doctor. (Community Pharmacist 2)
B4. External policies and incentives	Statement 8	We can do some collaborations between the private clinics, private pharmacies and government … pharmacists can do referral to private setting first … if the private clinic cannot solve this, then we refer to the public setting. (Community Pharmacist 14)
	Statement 9	We still have bills to pay, and we still have people under our employment to pay their salary. So, we need to have some form of reimbursement or support from the government for us to be able to do that. Because I believe a lot of community pharmacists have the heart for people, to reach out to people. It’s just money takes a huge chunk of that away from them. (Community Pharmacist 20)
Intervention characteristics		
C5. Relative advantage	Statement 10	For the TB patients, actually it’s quite time consuming and it’s not convenient for them to go to hospital for the DOT … It will save time to get that service and that is very convenient because community pharmacies are around. And then, the most important thing is that we can encourage them to be responsible to their own health. Then, we have more time to keep an eye on the patient also. (Community Pharmacist 13)
C6. Complexity	Statement 11	The concern maybe in the beginning … public will be scared of this idea because …TB patients [can] spread the disease to other people. But I think after a while … it will be accepted. (Community Pharmacist 17)
	Statement 12	We have to educate [the public] regarding how TB will spread … what precaution they can take and also what we can do to prevent the spread. I think with proper education, they can accept it. (Community Pharmacist 13)
C5. Relative advantage	Statement 13	If we do [TB-DOT] in community pharmacy… maybe this family has got TB, they have been following up with us for this six months, they will feel like, oh, I feel confident in this pharmacist. So next time, if I have anything need help, I will surely visit this pharmacist … I will not go to other place. Because this pharmacist has helped me to go through this period. This is vision … I’m very supportive, these kind of services. This is our professions. (Community Pharmacist 14)
	Statement 14	If we can make TB, a very common term to use, we introduce TB to the public. They feel TB, I know what is this. There is medication to control this, and I don’t have be worried … and they will not be afraid. And we will change the common stigma on this. (Community Pharmacist 14)
C7. Adaptability	Statement 15	I will say generally moderate, but it will be a bit higher for community pharmacists who are previously working together with a hospital setting or clinic setting. If they have an experience of working in multiple settings, the awareness might be higher. But if they started their career purely as in a community setting, maybe the awareness is moderate. And maybe there will be lack of experience with dealing with the TB medicine, because it is rare for us to dispense the medicine in a community setting for TB patients. (Community Pharmacist 9)
Characteristics of individuals		
D8. Knowledge and beliefs about the interventions D9. Self-efficacy	Statement 16	We rarely have any continuing medical education, CME talks on TB, lung health is quite rare. We have, we have a lot of CME on other topics, but not for tuberculosis … I think it is important pharmacist can do a lot to help TB patient. I think it’s important for a structured CME on how we can help TB patients navigate their disease. (Community Pharmacist 7)
	Statement 17	I think need to have like, training … a yearly thing, or just a one time off thing, in terms of like getting accreditation and certification, like the smoking cessation therapy … make sure we are equipped with the knowledge … and get accreditation or certification before providing such service. (Community Pharmacist 4)

CFIR = Consolidated Framework for Implementation Research; TB = tuberculosis; TB-DOT = tuberculosis-directly observed therapy.

#### Inner setting

The participants were concerned about the availability of manpower to provide TB-DOT, especially during peak hours and shift handover. To address this, they suggested scheduling appointments with TB patients to ensure they receive adequate monitoring and support during TB-DOT (Statement 1). Moreover, they believed a safe space should be set up to administer TB-DOT. This could be a private consultation room or a designated entrance and exit for TB patients (Statement 2).

In circumstances where there were constraints on manpower or space to build a private consultation room, participants were more receptive to the idea of providing TB-DOT through the drive-through service compared to vDOT. They were concerned about patients’ commitment to participate in vDOT where some might not be tech-savvy while some might have issues with internet connectivity (Statement 3).

In addition to TB-DOT, the participants agreed community pharmacists could contribute significantly in health promotion on TB. They could collaborate with the public health sector for public health campaigns to enhance public awareness on TB (Statement 4).

Interestingly, the participants were confident their employers would be supportive of adopting DOTS to improve the health outcomes of TB patients. The participants and their employers believed community pharmacy-based TB interventions could enable them to expand their professional role in TB management. This allows them to focus on patient-centred care to support patients’ treatment in primary care settings.

The positive treatment outcomes resulted from good patient-provider engagement could then improve the reputation of the pharmacy, which would likely be able to contribute to customer loyalty (Statement 5).

#### Outer setting

In terms of TB treatment, participants who had experience interacting with TB patients reported numerous challenges that TB patients encountered during their treatment process. The most observed barrier was non-adherence to TB medications due to a lack of understanding on the duration of treatment, pill burden, treatment side effects, and logistic difficulties. They felt that close supervision by community pharmacists who are just a stone’s throw away from TB patients could help improve their treatment adherence and health outcome (Statement 6 and Statement 7).

In addition to regulations, guidelines, and audits, they also believed a policy is needed for them to establish a strong collaboration with doctors to facilitate the transition of care for TB patients (Statement 8). The participants also expected reimbursement, remuneration, or incentives from the Government for their professional service and to cover the costs for personal protective equipment while providing TB-DOT to TB patients (Statement 9).

#### Intervention characteristics

The participants agreed that community pharmacy-based TB-DOT provides better accessibility, time flexibility, and convenience to TB patients in fulfilling the daily TB-DOT requirements. Moreover, community pharmacists are approachable where patients can easily engage with them for additional medical advice. These could help to ensure treatment adherence among patients (Statement 10). However, the participants also shared their concerns on stigma for community pharmacy-based TB-DOT, especially in the early stages of implementation. This is because the public generally has a low awareness and negative perception on TB. As mentioned, infection control and public education are essential in the efforts to destigmatize TB (Statement 11 and Statement 12). Therefore, community pharmacy-based TB-DOT service would enable pharmacists to contribute to treatment adherence among TB patients, build rapport with patients for customer loyalty, and improve public’s awareness on TB (Statement 13 and Statement 14).

The participants felt that community pharmacists who had working experience in the Government healthcare facilities would be better equipped with the skills to provide TB-DOT service at primary care settings. This is because TB cases are mainly managed in the Government-funded hospitals and clinics where pharmacists would have had the exposure of serving TB patients (Statement 15).

#### Characteristics of individuals

As participants had limited exposure, experience, and confidence in managing TB cases, they welcomed trainings to enhance their knowledge on TB management. They also felt that accreditation was important to increase confidence among TB patients and the public towards pharmacists’ competence in implementing DOTS programme (Statement 16 and Statement 17).

### Triangulation of data

Findings from the qualitative study were agreeable with the quantitative data where the participants acknowledged the benefits of community pharmacy-based TB-DOT. Several concerns were also raised with proposed suggestions to ensure the practicality of this intervention. The comparison of quantitative and qualitative data according to the CFIR domains was narrated as follows.

- *Inner setting*: Factors such as the availability of manpower and designated areas for TB-DOT might be the limitations for some pharmacies. Delivery of TB-DOT using the drive-through service was a considerably acceptable alternative for the participants. Qualitative data showed more positive response on employers’ support to expand the role of community pharmacists in adopting TB service at their pharmacies.
- *Outer setting*: Participants acknowledged the gaps in TB management, in particular non-adherence to TB treatment. Qualitative data indicated while community pharmacists have the potential to contribute to TB management, there is a need of policy and incentive schemes to strengthen the public-private partnerships.
- *Intervention characteristics*: In addressing the common concern on stigma against TB, qualitative findings found that community pharmacy-based TB-DOT should be viewed as an opportunity to engage with TB patients and the public, where pharmacists could help clearing up the misconceptions towards TB in the community.
- *Characteristics of individuals*: As participants lacked confidence and experience in managing TB, they believed trainings, accreditation, and support from the Government are essential prior to the implementation of TB service at community pharmacies.

## Discussion

This study highlighted the potential of DOTS programme as a feasible TB intervention at community pharmacies in Malaysia. Community pharmacists are regarded as highly trustable healthcare professionals and are easily accessible to members of community. The scope of pharmacy practice continues to expand and incorporates various pharmaceutical services that focus on patient-centred care. Participants in this study believed that community pharmacy-based DOTS programme could further expand their professional role in primary care. For example, a recent review reported that community pharmacists have the potential to improve TB outcomes with their role in TB screening, referrals, and TB-DOT (Wong *et al*., 2022).

While the Malaysia National TB Control Strategy Plan 2015 to 2022 indicated the importance of PPM in tackling TB, the role of community pharmacists was not explicitly outlined in the strategy plan (Ministry of Health Malaysia, 2016). In order to address the gaps in TB case detection and treatment outcomes, it is important to consider the incorporation of community pharmacists as an indispensable ally in the National TB Control programme. As suggested in the findings, community pharmacists established good rapport with their patients. This drove trust in patients and empowered them to work towards their health goals with the pharmacists to improve treatment outcomes. Indeed, studies that evaluated the impact of community pharmacist-led TB-DOT showed improvements in patients’ treatment completion (Antunes *et al*., 2012; Juan *et al*., 2006). In addition, as pharmacists interact with TB patients daily for TB-DOT, this rapport could also contribute to customer loyalty which drives profit to the pharmacy. The study by Guhl and colleagues has shown that personal interaction was one of the most significant factors that contributed to perceived customer value and customer loyalty for community pharmacies (Guhl *et al*., 2018).

Nevertheless, the concern on knowledge gap has been raised by the participants in this study. The lack of knowledge, experience, and confidence among community pharmacists coupled with public stigma against TB could be the challenge for community pharmacy-based DOTS programme. There is a need for training and education to ensure community pharmacists are equipped with adequate knowledge and experience prior to the implementation of pharmaceutical care services at community pharmacies (Loh *et al*., 2021). In addressing public stigma against TB, the study by Balogun and colleagues highlighted the impact of community health education on TB which had successfully improved public’s knowledge towards TB and reduced the stigma against TB (Balogun *et al*., 2015). As shown in the findings of this study, community pharmacists could expand their role in providing public education through campaigns in enhancing public awareness on TB.

To address issues on the limitations of space and manpower for TB-DOT at community pharmacies, the use of modified DOT, such as vDOT or drive-through service, received a mixed reaction. While several studies had found vDOT to improve treatment adherence among TB patients (Garfein *et al*., 2018; Sekandi *et al*., 2020; Story *et al*., 2019), the findings of this study showed that pharmacists had concern on patients’ commitment to attend vDOT. Therefore, in-depth counselling for TB patients and their caregivers must be conducted before their enrolment into vDOT, to ensure they strictly adhere to the vDOT appointment. As there was no study on TB-DOT through drive-through service that could be identified to date, research on this topic could be useful to evaluate its feasibility, practicality, and impact.

This study offers several strengths. This is the first study in Malaysia which introduced the concept of engaging community pharmacists in the DOTS programme. Moreover, a validated implementation science framework, the CFIR, was used to develop this study, which allows continuous guided evaluation of the intervention for improvement. However, this study needs to be interpreted considering the limitations. Since convenience sampling was used in this study, there was potential risk of sampling bias. While the minimum sample size for this study was achieved, it was noted that respondents were of younger age and most were serving at the urban areas. Therefore, the findings might not be generalizable to pharmacists who are more senior in age and those serving in suburban or rural areas. As details of the demographic and practice characteristics for community pharmacists in Malaysia were unavailable, the sample representativeness could not be compared. One of the demographic information on the states where the participants located was not collected from the participants. This was to avoid prejudice towards targeted states and locations in the country. Self-selection bias could also be resulted when pharmacists who had stronger interest in TB and pharmacy practice might have higher likelihood to respond and complete the survey and participate in the interview. While this was a self-reported study, there could be potential risk of desirability bias, where the respondents might answer the questions in a way which would be viewed more socially acceptable to others. Another limitation to note was the reliability test for the questionnaire was not performed. While this study aimed to understand the feasibility and practicality of the community pharmacy-based TB-DOT, a more pragmatic approach was taken with content and face validity assessments for the questionnaire before study recruitment. In the study recruitment, a total of 6330 study invitations, including reminders, were sent via emails. In addition, 500 paper copies of study invitations were sent by post. As the study invitations and reminders were also shared through electronic newsletters and social media platforms, the total number of questionnaires distributed could not be determined. Since this was the initial step to analyse the acceptability of community pharmacists towards community pharmacy-based TB intervention in Malaysia, detailed components such as costings and medication supplies were not discussed. These would be studied following the engagement sessions with the key stakeholders.

## Conclusion

This study highlighted the potential for community pharmacy-based TB-DOT service to be implemented in Malaysia. Community pharmacists who participated in this study believed that TB intervention at community pharmacies could help to improve TB outcomes. While they showed readiness to engage in TB-DOT service, there were regulatory concerns. A strong partnership with continuous engagement between the pharmacists and the Government needs to be established to develop an effective implementation plan to facilitate the decentralization of TB-DOT service at community pharmacies. This is to ensure an accessible and quality TB care to be provided for all levels of communities in the primary care settings.

## Supplementary material

For supplementary material accompanying this paper visit http://doi.org/10.1017/S1463423623000105. click here to view supplementary material
