# Supplementary material for: Engaging community pharmacists in tuberculosis-directly observed treatment: a mixed-methods study
Source: Prim Health Care Res Dev. 2023 Mar 22;24:e21. doi: 10.1017/S1463423623000105 (PMC10050952; doi:10.1017/S1463423623000105)
Supplement: Supplementary file 1 [file S1463423623000105sup001.docx]

**Title: Engaging Community Pharmacists in Tuberculosis Management: A Mixed Methods Study**

**Supplementary Material**

Contents

[Supplementary material S1 2](#_Toc115862470)

[Supplementary material S2 3](#_Toc115862471)

[Supplementary material S3 10](#_Toc115862472)

[Supplementary material S4 11](#_Toc115862473)

## Supplementary material S1

Table S1 Published literature used as reference for developing the study questionnaire and interview topic guide

| Author | Title |
| --- | --- |
| Aibana 2020 | Patients' perspectives of tuberculosis treatment challenges and barriers to treatment adherence in Ukraine: a qualitative study |
| Hurtig 2000 | Anti-tuberculosis treatment in private pharmacies, Kathmandu Valley, Nepal |
| Kobaidze 2009 | Over the Counter Availability of Antituberculosis Drugs in Tbilisi, Georgia in the Setting of a High Prevalence of MDR-TB |
| Meyerson 2020 | Feasibility and acceptability of a proposed pharmacy-based harm reduction intervention to reduce opioid overdose, HIV and hepatitis C |
| O'Reilly 2015 | A feasibility study of community pharmacists performing depression screening services |
| Rajeswari 2002 | Private pharmacies in tuberculosis control--a neglected link |
| Watson 2009 | The feasibility of providing community pharmacy-based services for alcohol misuse: a literature review |
| Wong 2022 | Community pharmacists-led interventions in tuberculosis care: A  systematic review |

## Supplementary material S2

**Project Title: Transition of Tuberculosis Care to Community Pharmacies (MUHREC: Project 27294)**

**Introduction:** Tuberculosis (TB) affects millions of people in the world. In Malaysia, TB is mainly managed by the public health service. In order to optimize health care resources for quality medical care, this opens an opportunity for us to look into the feasibility of Transfer of Care (ToC) for TB management from the public health facilities to community pharmacies. We hope to use the answers that you give us to help improve TB management through the public-private partnerships (PPP) in Malaysia.

Community pharmacies and community pharmacists are the first point of contact to many members of the communities who require medication advice and service. With the success of pharmacist-led interventions in managing medical conditions such as diabetes mellitus as well as smoking cessation and Methadone Replacement Therapy, we wish to explore the feasibility of community pharmacy-based TB service in Malaysia. This is not intended to be a compulsory service, but an additional value-added service to support the TB control and prevention programme. Here, we would like to know your perception, as a community pharmacist, about the delivery of TB service at community pharmacies.

**Consent form**

You are invited to participate in this study. You may access the participant information sheet by clicking and downloading this file: Explanatory statement

Do you agree to participate in the study? By clicking YES to ALL the statements, you are giving consent that you are willing to answer the questions in this survey.

I have been asked to take part in the Monash University research project specified above. I have read and understood the Explanatory Statement and I hereby consent to participate in this project (online survey questionnaire).

- I agree
- I disagree [to the end of the questionnaire]

**Part 1: Demographic information**

We would like to know your basic demographic information. This information will only be used to describe the demographics of respondents who answer and complete this survey.

1. Please indicate your age:

__________

1. Please indicate your gender:

__________

1. When did you obtain the Fully Registered Pharmacist (FRP) Certificate? (year):

__________

1. How long have you been practicing as a community pharmacist? (year):

__________

1. Have you been certified to provide specialized pharmacotherapy services?

- Yes
- No

1. Did you have experience working in the Government hospital or health clinic?

- Yes
- No

**Part 2: Pharmacy settings**

We would like to know the characteristics of the community pharmacy you work at, to better understand the facilitators and limitations to TB service at community pharmacies.

1. Please indicate the location of the community pharmacy you work at*:

- urban
- suburban
- rural

* The definitions of rural, suburban and rural locations in the Malaysian are based upon the definitions obtained from the Statistics Department of Malaysia (2020):

- Urban: The area has a population of 10,000 or more
- Rural: Areas other than urban locations, comprising of all types of settlement villages, small towns and other settlements of less than 10,000 populations, with agricultural characteristics and rich in natural resources
- Suburban: Town or a small settlement with urban characteristics located in rural areas with less than 10,000 population, accommodating small scale commercial activities, public facilities and weekly activities (morning/agriculture markets, day/night markets)

1. Please indicate the setting of the community pharmacy you work at:
   - Shop lot
   - Shop complex (shopping mall)
   - Health care facilities
   - Others, please specify:
2. What is the ownership status of this community pharmacy?
   - Independent (Single proprietor / Partnership)
   - Franchise
   - Chain
   - Others, please specify:
3. On average, how many customers or clients does your pharmacy serve in a day?

__________

1. How many full-time fully registered pharmacists work at this community pharmacy per shift?

__________

1. How many pharmacy assistants and staff work at this community pharmacy per shift?

__________

1. Does this community pharmacy have a private consultation room?

- Yes
- No

1. Does this community pharmacy provide specialized pharmacotherapy services (for example smoking cessation therapy, diabetes management, compounding etc)?

- Yes
- No

**Part 3: Tuberculosis**

In the following session, we would like to know briefly about your understanding on tuberculosis (TB). Please rest assured that this is not an assessment to gauge your knowledge, we would be very grateful if you could respond to the questions honestly.

1. To your knowledge, is there a National Tuberculosis Control Programme (NTP) in Malaysia?

- Yes
- No

1. What are the symptoms of active pulmonary TB? (Select all that apply)

- Prolonged cough (more than 2 weeks)
- Haemoptysis
- Gastric pain
- Night sweat
- Loss of weight
- Fever

1. What are the regimens used in treating active pulmonary TB in Malaysia according to the Good Clinical Practice? (Select all that apply)

- Rifampicin
- Daptomysin
- Streptomycin
- Ethambutol
- Pyrazinamide
- Isoniazid

1. What are the side effects related to TB medications? (Select all that apply)

- discolouration of urine
- blurred vision
- hepatotoxicity
- joint pain

1. At this point of time, do you know what to do when a TB patient experiences side effects due to TB medications?

- Yes
- No

1. Based on your experience, what are the potential difficulties or challenges that TB patients face? (Please rank each of the following options in order of importance with #1 being the most important challenge to be addressed, to #10 being the least important challenge to be addressed). RANK:

- Not adherent to medication consumption
- Experiencing side effects to medications
- Difficulty to go hospital or health clinic daily for TB-DOT, due to time constraint
- Difficulty to go hospital or health clinic daily for TB-DOT, due to logistic issues
- Difficulty to follow-up with routine hospital or clinic appointments
- Unaware of the severity of TB disease
- Intimidated by the health care professionals' attitude
- Prolonged isolation
- Stigma and discrimination
- Others, please state:

1. Community pharmacists in Malaysia may have limited role in TB management. However, in your opinion, how could a community pharmacist contribute to TB management? (Select all that apply)

- Refer customers with suggestive symptoms of active pulmonary TB to doctors or specialists for thorough examination
- TB medication counselling
- Observe and witness patients or clients taking TB medications (TB-directly observed treatment, TB-DOT)
- Send reminder to TB patients to ensure their adherence in taking TB medications
- Review medication list of patients to identify any possible drug interactions
- Monitor side effects of the TB medications, refer patients to doctors or specialists when necessary
- Maintain records of visits by checking TB patients who are lost to follow-up
- Educate public and patients on tuberculosis prevention practice
- Others, please state:

1. Have you ever referred patients or customers with suggestive symptoms of active pulmonary TB for medical screening and examination from your community pharmacy for the past one year?

- Yes
- No

1. Do you think there is a need for an official and structured referral system for referral?

- Yes
- No

1. How often does a patient or customer look for TB medications at your community pharmacy for the past one year?

- More than 7 times a week
- 5 to 7 times a week
- 2 to 4 times a week
- Once a week
- Once every fortnight
- Once every month
- Never
- Others, please state:

1. Dispensing of TB medications at community pharmacies may not be a common practice in Malaysia. However, do you think there is a need of dispensing and re-filling TB medications at community pharmacies in Malaysia?

- Yes
- No

**Part 4: TB Service at Community Pharmacy**

The DOTS control strategy is a cost-effective approach to curb the spread of TB. It consists of five components: (1) government commitment, (2) case detection by sputum smear microscopy, (3) regular drug supply, (4) a standardized recording and reporting system, and (5) the directly observed treatment (TB-DOT) where TB patients take their daily medications under the supervision and observation of a qualified healthcare worker.

As a way to facilitate TB-DOT service at community pharmacy, we propose, TB-DOT to be provided to TB patients who have started TB treatment for more than 2 weeks (where most people are no longer infectious), they could either be discharged from the general hospitals or TB patients who are receiving TB-DOT at the Government hospital or health clinics, and wish to opt for community pharmacy-based TB-DOT.

1. TB-DOT at community pharmacies has not been implemented at community pharmacies in Malaysia. However, in your opinion, do you think there is a need for TB-DOT at community pharmacies in Malaysia?

- Yes
- No

1. Are you willing to introduce and provide TB-DOT to TB patients or customers who may need it?

- Yes
- No

1. If yes, you would be happy to provide TB-DOT for TB patients in which phase of TB treatment? Note: Drug sensitive TB disease treatment regimen consists of an initial 2-month intensive phase followed by a maintenance phase of at least 4 months.

- Intensive phase only (at least 2 weeks after the initiation of TB treatment, when it is not contagious)
- Maintenance phase only
- Both intensive and maintenance phases

1. At this point of time, are you confident to provide TB-DOT at community pharmacies?

- Yes
- No

1. In your opinion, how would TB-DOT at community pharmacies benefit the TB patients? (Select all that apply)

- Less traveling time
- Lesser waiting time spent to receive TB- DOT
- Time flexibility
- Less crowded
- Better accessibility
- Improve treatment adherence / less dropout
- Reduce the risk of multidrug-resistant TB (MDR-TB)
- Close monitoring of treatment side effects
- Promote patient-centred care
- Improve treatment success
- Improve patient engagement, leading to better trust and confidence in community pharmacists
- Others

1. In your opinions, do you think delivering TB-DOT at community pharmacies could put the health of community pharmacists, pharmacy staff and customers at risk, considering the risk of an airborne disease?

- Yes
- No

1. In your opinion, do you think your pharmacy needs to be upgraded to accommodate the delivery of TB-DOT, for example to establish a private consultation room, counter shield etc?

- Yes
- No

1. Are you willing to invest in additional special kits or Personal Protective Equipment (PPE) such as the N95 masks to deliver TB-DOT?

- Yes
- No

1. What are the potential concerns you have towards DOTS at community pharmacies? (Please rank each of the following options in order of the degree of concern, with #1 being the most concerned barrier, to #10 being the least concerned barrier.

- Community pharmacists would be overly burdened by the workload of delivering TB-DOT
- Delivering TB-DOT could be very time consuming for community pharmacists
- Unsure whether training and support will be provided and readily available from the stakeholders or partnership
- Unsure about the regulation requirements
- Stigma and pharmacy sales affected
- Community pharmacists are not interested to provide TB-DOT
- Lack of reimbursement, remuneration or incentives
- Others, please state:

1. Do you think TB-DOT at community pharmacies is too complicated to be implemented?

- Yes
- No

1. It is acknowledged that only a limited number of community pharmacies in Malaysia provide drive-through service, due to substantial restrictions. Nevertheless, in your opinion, do you think that providing TB-DOT by drive-through could be a practical alternative?

- Yes
- No

1. In your opinion, do you think that monitoring TB-DOT through video call (video DOT) by community pharmacists could be a practical alternative?

- Yes
- No

1. If you are an employee to your community pharmacy, do you think your employer would be supportive to implement DOTS (including TB-DOT) as part of community pharmacists' daily practice?

OR

If you are an employer, would you be supportive to implement DOTS (including TB-DOT) as part of community pharmacists' daily practice?

- Yes
- No
- Unsure

1. Do you think community pharmacists should receive reimbursement, remuneration or incentives for providing DOTS?

- Yes
- No

1. Based on your opinion, what aspects of the TB service at the community pharmacies are likely to be audited? (Select all that apply)

- Administrative measures
- Environmental measures
- Infection control
- Training of the staff on infection control
- Staff personal protective respiratory measures
- Treatment outcomes
- Management of adverse events from TB treatment
- Others, please state:

1. How effective do you think audit for TB service at community pharmacies is able to improve the quality of care and treatment adherence of TB patients?

- Extremely effective
- Very effective
- Moderately effective
- Slightly effective
- Not effective at all

1. If DOTS is to be implemented at your community pharmacy, how willing are you to be committed to audit for monitoring and regulations in providing TB service?

- Extremely willing
- Very willing
- Moderately willing
- Slightly willing
- Not willing at all

This is the end of the survey. Thank you for your time.

## Supplementary material S3

In the year of 2021 when the study was conducted, there were approximately 6,000 pharmacists practising in the private sector in Malaysia. The following sample size formula was used for the sample size calculation:

$$n=N x \frac{\frac{Z^{2}x p x (1-p)}{e^{2}}}{[N-1+ \frac{Z^{2} x p x \left( 1-p \right)}{e^{2}}]}$$

| N | Population size | 6,000 |
| --- | --- | --- |
| Z | Z-score in response to 95% confidence level | 1.96 |
| e | Margin of error | 5% |
| N | Sample proportion | 0.5 |
| n | Ideal sample size | 362 |

We accounted for 5% of drop-out (Dong and Peng, 2013; Schafer, 1999), therefore a minimum of 380 participants was required for recruitment.

## Supplementary material S4

Table S4 Topic guide

| Question | Topic guide | CFIR domain |
| --- | --- | --- |
| 1 | What do you know about TB? What is your perception about TB? In your opinion, how well is Malaysia managing TB? | Characteristics of individuals |
| 2 | What were your experiences with TB patients? In your opinion, what are the potential difficulties or challenges that TB patients face? | Outer setting |
| 3 | What and how was your exposure towards TB? How confident you are in managing TB cases? | Characteristics of individuals |
| 4 | Community pharmacists in Malaysia may have limited role in TB management. However, in your opinion, how could a community pharmacist contribute to TB management? How supportive would your employers be? | Inner setting;  Outer setting |
| 5 | How do you think community pharmacy-based TB service and TB directly observed treatment (TB-DOT) can benefit TB patients? | Intervention characteristics |
| 6 | What are your concerns and barriers towards community pharmacy-based TB service and TB-DOT? What suggestions do you have in addressing the said concerns? | Inner setting;  Intervention characteristics;  Characteristics of individuals |
| 7 | How willing are you to introduce and provide TB-DOT to TB patients or customers who may need it? | Intervention characteristics |
| 8 | What policy and incentives do you think is needed for this intervention? | Outer setting |
| 9 | Do you have additional comments to share with us? |  |
